# Supplementary material for: TERT expression is associated with metastasis from thin primaries, exhausted CD4+ T cells in melanoma and with DNA repair across cancer entities
Source: PLoS One. 2023 Jul 7;18(7):e0281487. doi: 10.1371/journal.pone.0281487 (PMC10328343; doi:10.1371/journal.pone.0281487)
Supplement: S1 File — (DOCX) [file pone.0281487.s001.docx]

# Supplementary Material for

## *TERT* expression is associated with metastasis from thin primaries, exhausted CD4+ T cells in melanoma and with DNA repair across cancer entities

## Supplementary methods

### Transcriptomics

We analyzed several melanoma bulk RNA-seq cohorts ([Table 1](https://docs.google.com/document/d/1LAyORUyfW_o7pELrfAkZ6qLYoMUt4viKLpYj5stPirw/edit#heading=h.c29pmhl2rpsc)): SKCM [[1]](https://paperpile.com/c/vLfZxA/0xxOg), Gide [[2]](https://paperpile.com/c/vLfZxA/k99oX), Hugo [[3]](https://paperpile.com/c/vLfZxA/l3HOs), Liu [[4]](https://paperpile.com/c/vLfZxA/xxLJj), Riaz [[5]](https://paperpile.com/c/vLfZxA/YRihg), Van Allen [[6]](https://paperpile.com/c/vLfZxA/7VQA7) and one cohort across-cancer types (pan-cancer analysis of whole genomes PCAWG [[7]](https://paperpile.com/c/vLfZxA/ut2HZ)). Furthermore we included the cancer cell line encyclopedia CCLE [[8]](https://paperpile.com/c/vLfZxA/qtLUQ), a melanoma single-cell RNA-seq dataset [[9]](https://paperpile.com/c/vLfZxA/nRyJM), the MSK IMPACT Clinical Sequencing Cohort (MSKCC, Nat Med 2017, [[10]](https://paperpile.com/c/vLfZxA/Fu2jB)) with corresponding therapy data [[11]](https://paperpile.com/c/vLfZxA/snUWd) and a glioblastoma bulk tumor cohort [[12]](https://paperpile.com/c/vLfZxA/1mZWR) in our workflow (S1 Fig).

We quantified gene expression from fastq files in transcripts per million mapped reads (TPM) per gene using kallisto [[13]](https://paperpile.com/c/vLfZxA/FF4Es) for the Gide (PRJEB23709 at ENA), Riaz (GSE91061), Hugo (SRR3184279-SRR3184306) and Van Allen cohorts. The annotation of transcripts to genes was performed using the Homo_sapiens.GRCh38.96.gtf as provided by kallisto. Quantifications of gene expression were used as published for the other cohorts: SKCM, Liu, PCAWG, CCLE, Jeby-Arnon, and Klughammer.

The quantification of isoforms from the raw sequencing data (fastq files) for the Van Allen cohort was performed using kallisto version 0.45.1 on Ensembl v96 transcriptome. The quantifications of isoforms for the PCAWG tumors and the CCLE cell lines were obtained at <https://dcc.icgc.org/releases/PCAWG/transcriptome/transcript_expression/pcawg.rnaseq.transcript.expr.tpm.tsv> and <https://portals.broadinstitute.org/ccle/data>. As the annotation of isoforms for the three cohorts differs, we identified differential isoforms in the datasets separately. We then used the isoform identifiers and investigated them with respect to their structure and exon usage for the analysis of the mitochondrial targeting sequence.

### *TERT* isoform information

We quantified six isoforms, three of which were most highly expressed in healthy tissue (mainly in testicular tissue, Gtex database, S2 Fig): TERT-201, TERT-204, TERT-205 (ENSEMBL isoform IDs see Methods) and three isoforms: TERT-008, TERT-202, TERT-203. TERT-201 comprises all 16 exons and is reported to have full catalytic activity. The two other prominent isoforms, TERT-204, lacking exon 1, 2, 7, and 8 and TERT-205, which consist of only the two exons 10 and 11, are both catalytically inactive isoforms. In the presence of a *TERT* promoter mutation 3 of 6 tested isoforms were consistently upregulated in at least two of three cohorts [(](https://docs.google.com/document/d/1GTD4DBABzvett1BH1WzCUPCjsbx9Jzd91TMTbw4t96M/edit#heading=h.1ci93xb)S8A Table, S8 Fig). The three up-regulated ENSEMBL isoforms were: the full-length isoform TERT-201 and the two catalytically inactive isoforms TERT-202 and TERT-203. However, in the PCAWG cohort all *TERT* isoforms were up-regulated with *TERT* promoter mutations. Therefore, it remains unclear whether the observed increased isoform expression was the result of alternative splicing due to mutations of the *TERT* promoter at the transcription start site or a result of a general increase in *TERT* expression.

Because of lacking exon 1, TERT-204 and TERT-205 also lack the mitochondrial targeting sequence. TERT-008, TERT-202 and TERT-203 are also catalytically inactive isoforms but include the mitochondrial targeting sequence. To determine the proportion of isoforms with mitochondrial targeting sequence, we used the formula: ([,'with_mito_leader'])/([,'with_mito_leader']+[,'without_mito_leader'])==TRUE; with_mito_leader=[,'TERT_ENST00000310581']+[,'TERT_ENST00000334602']+[,'TERT_ENST00000460137']+[,'TERT_ENST00000522877']; without_mito_leader=[,'TERT_ENST00000484238']+[,'TERT_ENST00000484238'] at https://tools.hornlab.org/cru337phenotime/.

### *TERT* promoter mutation calls

*TERT* promoter genotypes for the Van Allen cohort on positions (228, 242, 250) were called from exome bam files using bcftools call with an adjusted prior (-P 0.1). Samples which had at least 2 or more reads per position were kept for analysis. CCLE *TERT* promoter mutations were obtained from the supplement (<https://www.ncbi.nlm.nih.gov/pmc/articles/PMC6697103/bin/NIHMS1032762-supplement-Suppl_Table_5.xlsx>) of [[8]](https://paperpile.com/c/vLfZxA/qtLUQ). PCAWG *TERT* promoter mutations were obtained from the supplement (<https://static-content.springer.com/esm/art%3A10.1038%2Fs41467-019-13824-9/MediaObjects/41467_2019_13824_MOESM3_ESM.xlsx>) of [[14]](https://paperpile.com/c/vLfZxA/9Gx75). *TERT* promoter mutations for TCGA SKCM were obtained from the supplement (<https://www.ncbi.nlm.nih.gov/pmc/articles/PMC4580370/bin/NIHMS698912-supplement-3.xlsx>) of the study [[1]](https://paperpile.com/c/vLfZxA/0xxOg).

### Immune signatures

The quantification of immune infiltrates was carried out using [TIMER 2.0](http://timer.cistrome.org/) [[15]](https://paperpile.com/c/vLfZxA/WwGxp). For the six bulk RNA-seq melanoma cohorts (SKCM, Gide, Hugo, Liu, Riaz, Van Allen) expression data were uploaded in the immune estimation tab with the SKCM cancer type selected [[16]](https://paperpile.com/c/vLfZxA/4aVjj). These data were then added to the annotation table provided for each published cohort (see data availability).

##

## Supplementary tables (separately uploaded files)

[**S1 Table**](https://docs.google.com/spreadsheets/d/1YPkhc2XXs3rFKlwOv3oO9K0Fp68MbRxGf4owW6EWCDg/edit#gid=1174909472)**. Multivariate survival analysis.** Multivariate analysis using Cox proportional hazard models for progression free survival (PFS) (S1A Table) and overall survival (OS) (S1B Table) subsetted for ICI therapy type in cohorts Liu, Gide, Hugo, Riaz, Van Allen and SKCM. (Supp_Table_S1.xlsx)

[**S**](https://docs.google.com/spreadsheets/d/1CFJczz083argpIVHwSQ_nY-dqxCMGOMZ2oP7u11ciog/edit#gid=0)**2 Table.** ***TERT* expression and immune signatures.** Significant immune signatures in SKCM associated with *TERT* expression and comparison against other bulk RNA-seq cohorts (Gide, Hugo, Liu, Riaz, Van Allen).

(Supp_Table_S2.xlsx)

**S3 Table.** **TERT with respect to clinical variables.** Wilcoxon tests for group comparisons and Spearman correlations of clinical variables against *TERT* expression and *TERT* promoter mutation in Liu, SKCM and PCAWG cohorts (S3A-E Tables). (Supp_Table_S3.xlsx)

**S4 Table. TERT in melanoma subtypes.** Mutation subtypes against *TERT* expression (S4A Table) and *TERT* promoter mutation status (S4B Table) in the SKCM melanoma cohort. Effect of hotspot mutations on *TERT* expression in *TERT* promoter mutated samples (S4C Table). (Supp_Table_S4.xlsx)

[**S**](https://docs.google.com/spreadsheets/d/1XZy_5-bTBmv-iddi_8InouJoxrDlMrc25yynNYgUz3Y/edit#gid=1788848893)**5 Table**[**.**](https://docs.google.com/spreadsheets/d/1XZy_5-bTBmv-iddi_8InouJoxrDlMrc25yynNYgUz3Y/edit#gid=1788848893) ***TERT* isoforms and mutation subtypes and influence of *TERT* alterations on ROS**. Influence of *TERT* promoter mutation on *TERT* isoforms, mitochondrial targeting sequence (S5A Table), associations of ROS-related genes with *TERT* expression levels (S5B Table), and *TERT* promoter mutation status (S5C Table) in Gide, Riaz, Hugo, Liu, Van Allen, SKCM, PCAWG, CCLE cohorts, and single-cell. Difference on *TERT* promoter mutation subtypes on *TERT* expression on SKCM and CCLE cohorts (S5D Table). (Supp_Table_S5.xlsx)

[**S**](https://docs.google.com/spreadsheets/d/1tkEzFDDjXVEFvfMdEwYPcColVzqv-8fZkV9vWTvrh-Y/edit#gid=753074891)**6 Table**[**.**](https://docs.google.com/spreadsheets/d/1tkEzFDDjXVEFvfMdEwYPcColVzqv-8fZkV9vWTvrh-Y/edit#gid=753074891) **Genetic correlates with *TERT* promoter mutations.** Genes associated with *TERT* promoter mutation status for the Van Allen, SKCM, PCAWG, and CCLE cohorts, and overlap of cohorts (S6A-E Tables). (Supp_Table_S6.xlsx)

[**S**](https://docs.google.com/spreadsheets/d/1uHISkQnJK0DcCLUB3ocbqH3VHXztQVjQhARUY1V-tWA/edit#gid=0)**7 Table. Genetic correlates with *TERT* expression.** Genes associated with *TERT* expression in each cohort (Gide, Riaz, Van Allen, Hugo, Liu, SKCM, PCAWG, CCLE, single-cell, and Klughammer) and overlap of cohorts (S7A-K Tables). (Supp_Table_S7.xlsx)

[**S**](https://docs.google.com/spreadsheets/d/1-0fV6UUqf6lp_NJi806tGwFMj6C1beva1euAT4hhK1w/edit#gid=1754710510)**8 Table**[**.**](https://docs.google.com/spreadsheets/d/1-0fV6UUqf6lp_NJi806tGwFMj6C1beva1euAT4hhK1w/edit#gid=1754710510) **Enrichment analysis.** Enrichment analysis of 17 genes associated with *TERT* expression and 30 genes associated with *TERT* promoter mutation in bulk tumors and cell lines (Supplementary Table S8A Table), and for the top 100 genes associated with *TERT* expression in single-cells (S8B Table). (Supp_Table_S8.xlsx)

## Supplementary figures


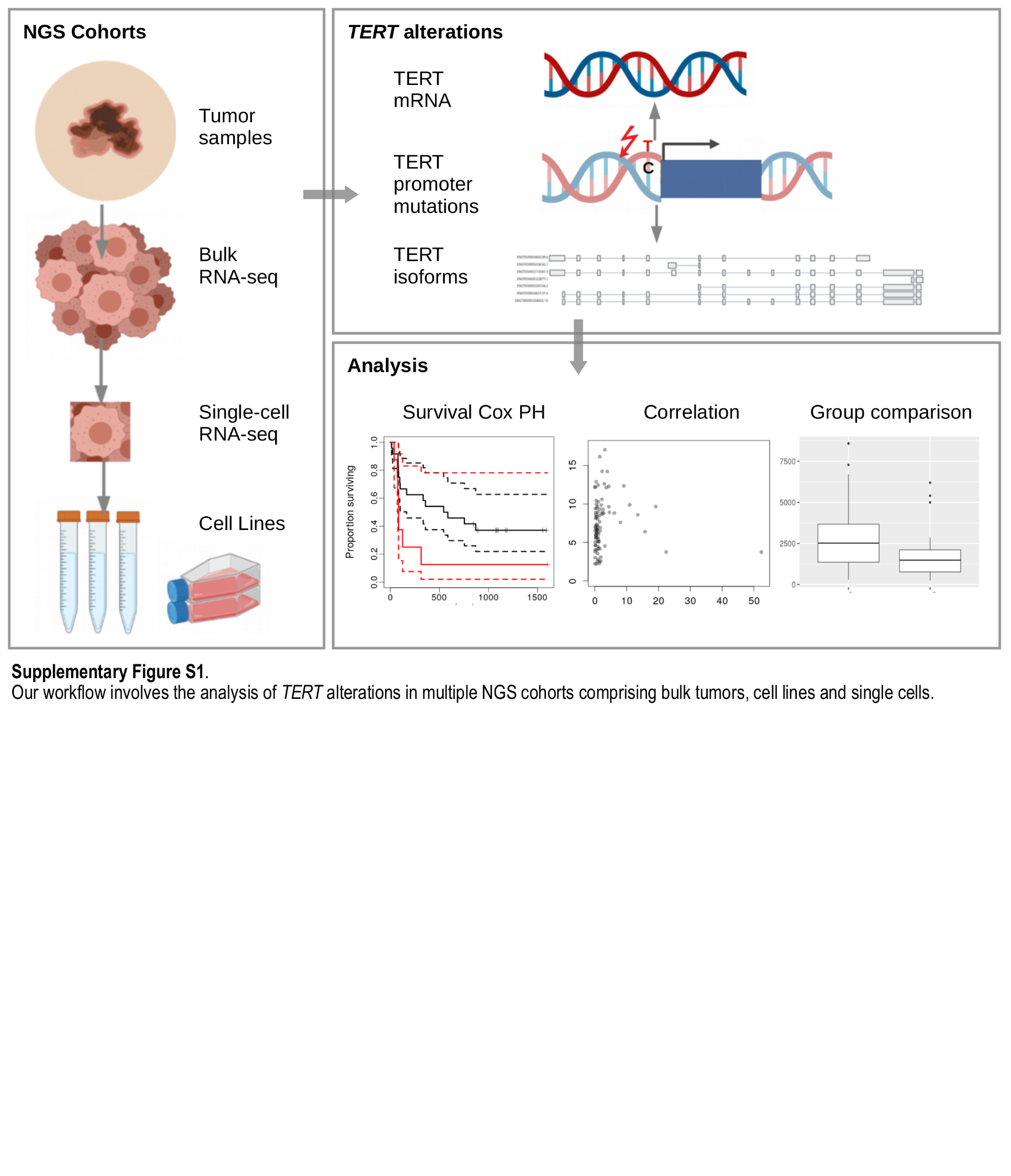


### S1 Fig. Overview of the analysis workflow. Our workflow involves the analysis of *TERT* alterations in multiple NGS cohorts comprising bulk tumors, cell lines and single cells.


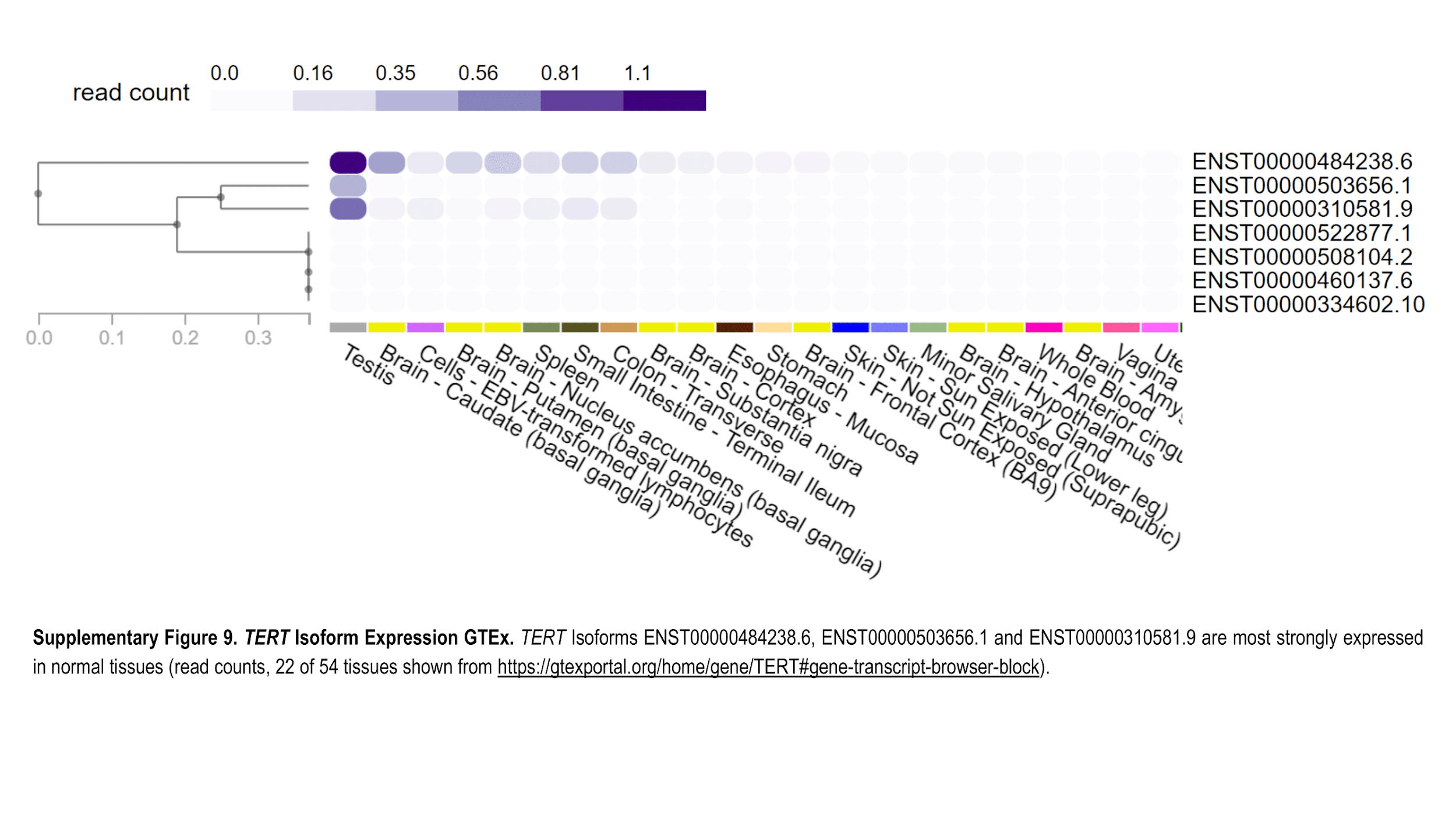


### S2 Fig. *TERT* isoform expression in normal control tissues (Getx database). *TERT* isoforms ENST00000484238.6 (TERT-204), ENST00000503656.1 (TERT-205) and ENST00000310581.9 (TERT-201) are expressed predominantly in testis (read counts, 22 of 54 tissues shown from<https://gtexportal.org/home/gene/TERT#gene-transcript-browser-block>).


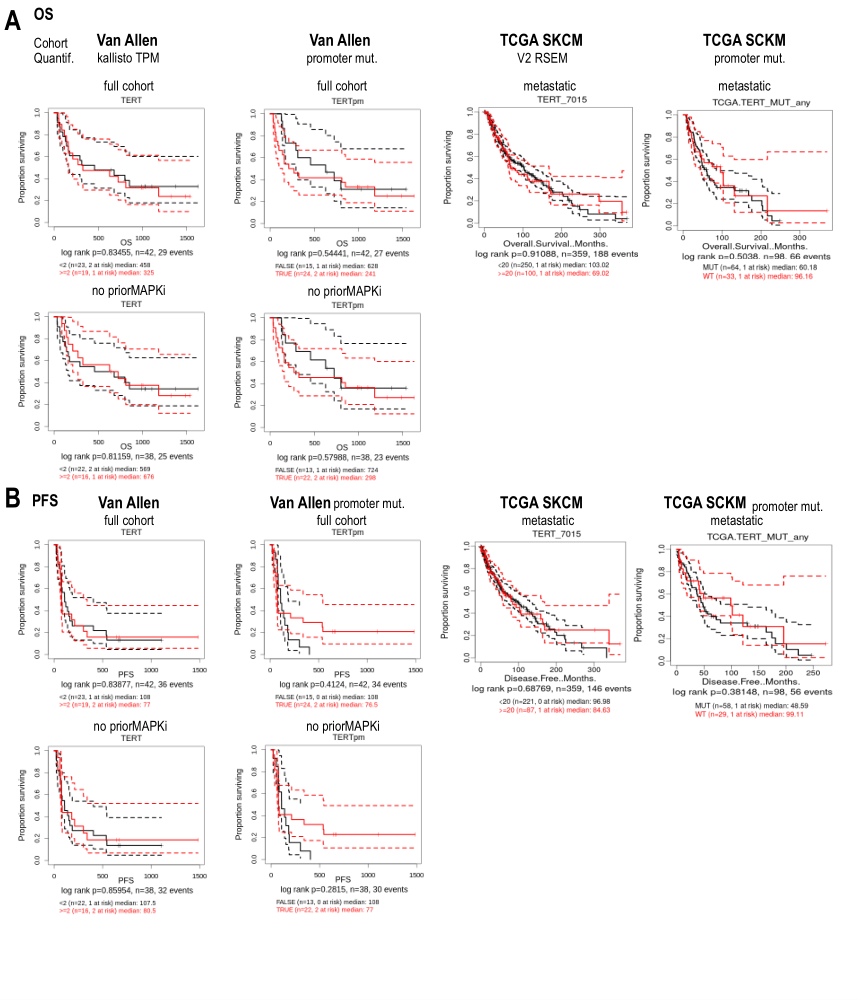


###

### S3 Fig. Survival under anti-CTLA4 ICI with ipilimumab (Van Allen) and in non-ICI melanoma (SKCM). Comparison of *TERT* low vs. high and *TERT* promoter mutated patients (based on signature:[,'TERTpm']==TRUE). (A) Patients with *TERT* genetic changes do not show altered overall (OS) and (B) progression free survival (PFS). Univariate p-values from log rank tests. Dashed: confidence intervals. Note the varying thresholds for *TERT* expression in each cohort is due to the classification that the patient group is approximately one third patients with high *TERT* expression. ICI: immune checkpoint inhibition; PFS: progression-free survival; priorMAPKi: prior therapy targeting mitogen-activated protein kinase pathway; OS: overall survival; SKCM: skin cutaneous melanoma; TERT: telomerase reverse transcriptase


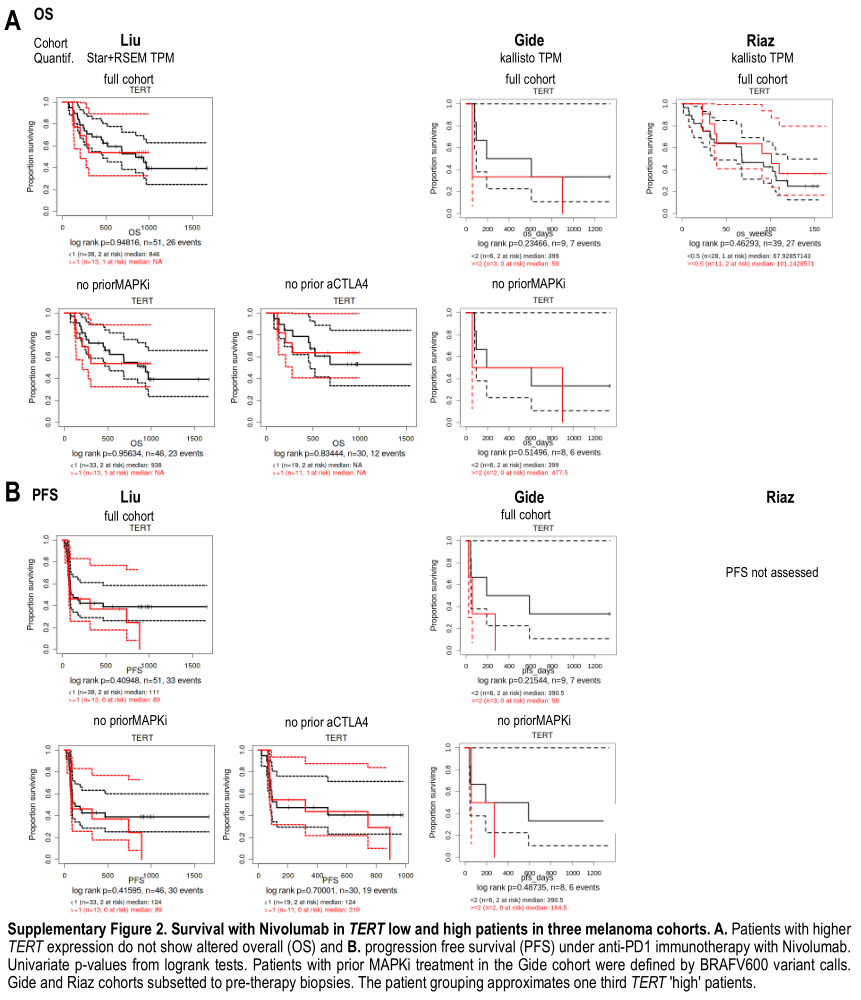


### S4 Fig. Survival under anti-PD1 ICI with nivolumab in three melanoma cohorts (Liu, Gide, Riaz). Comparison of *TERT* high vs. low patients. (A) Patients with higher *TERT* expression do not show altered overall (OS) and (B) progression free survival (PFS). Patients with prior MAPKi treatment in the Gide cohort were defined by *BRAF*V600 variant calls. Gide and Riaz cohorts subsetted to pre-therapy biopsies. Univariate p-values from log rank tests. Dashed: confidence intervals. Note the varying thresholds for *TERT* expression in each cohort is due to the classification that the patient group is approximately one third patients with high *TERT* expression. ICI: immune checkpoint inhibition; PFS: progression-free survival; priorMAPKi: prior therapy targeting mitogen-activated protein kinase pathway; OS: overall survival; SKCM: skin cutaneous melanoma; TERT*:* telomerase reverse transcriptase.


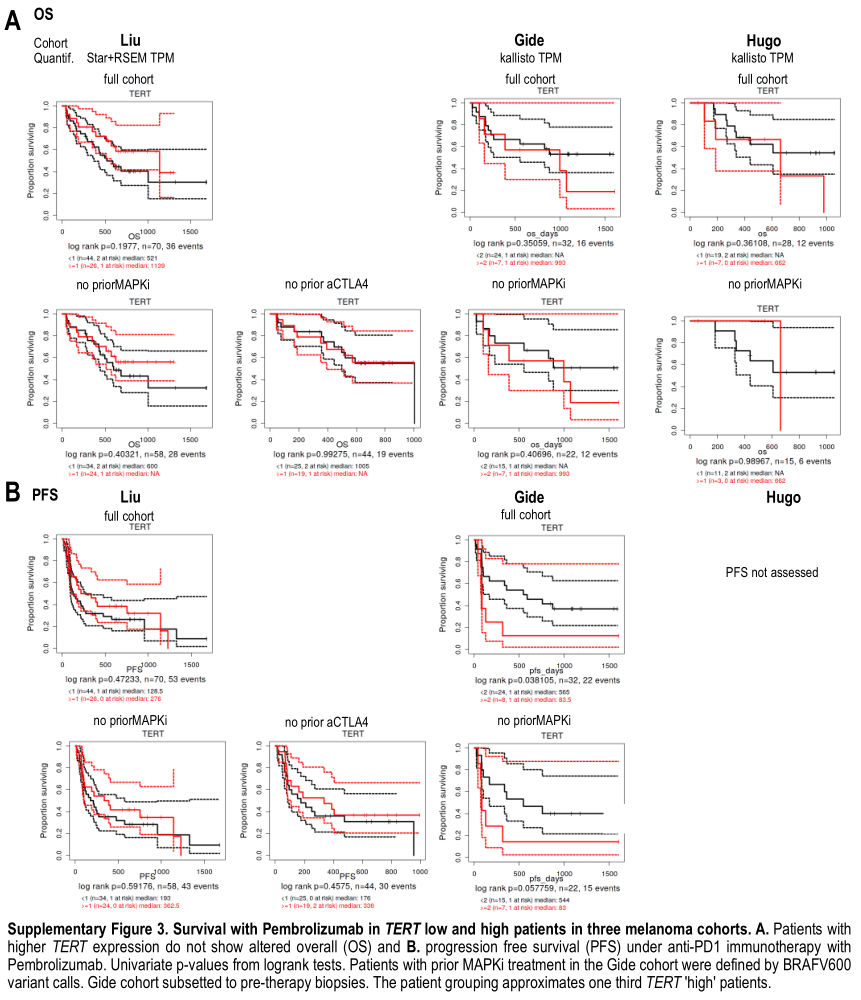


### S5 Fig. Survival under anti-PD1 ICI with pembrolizumab in three melanoma cohorts (Liu, Gide, Hugo). Comparison of *TERT* low vs. high patients. (A) Patients with higher *TERT* expression do not show altered overall (OS) and (B) progression free survival (PFS), except for the Gide cohort (p=0.038). Patients with prior MAPKi treatment in the Gide cohort were defined by *BRAF*V600 variant calls. Gide cohort subsetted to pre-therapy biopsies. Univariate p-values from log rank tests. Dashed: confidence intervals. Note the varying thresholds for *TERT* expression in each cohort is due to the classification that the patient group is approximately one third patients with high *TERT* expression. ICI: immune checkpoint inhibition; PFS: progression-free survival; priorMAPKi: prior therapy targeting mitogen-activated protein kinase pathway; OS: overall survival; SKCM: skin cutaneous melanoma; TERT*:* telomerase reverse transcriptase.

###
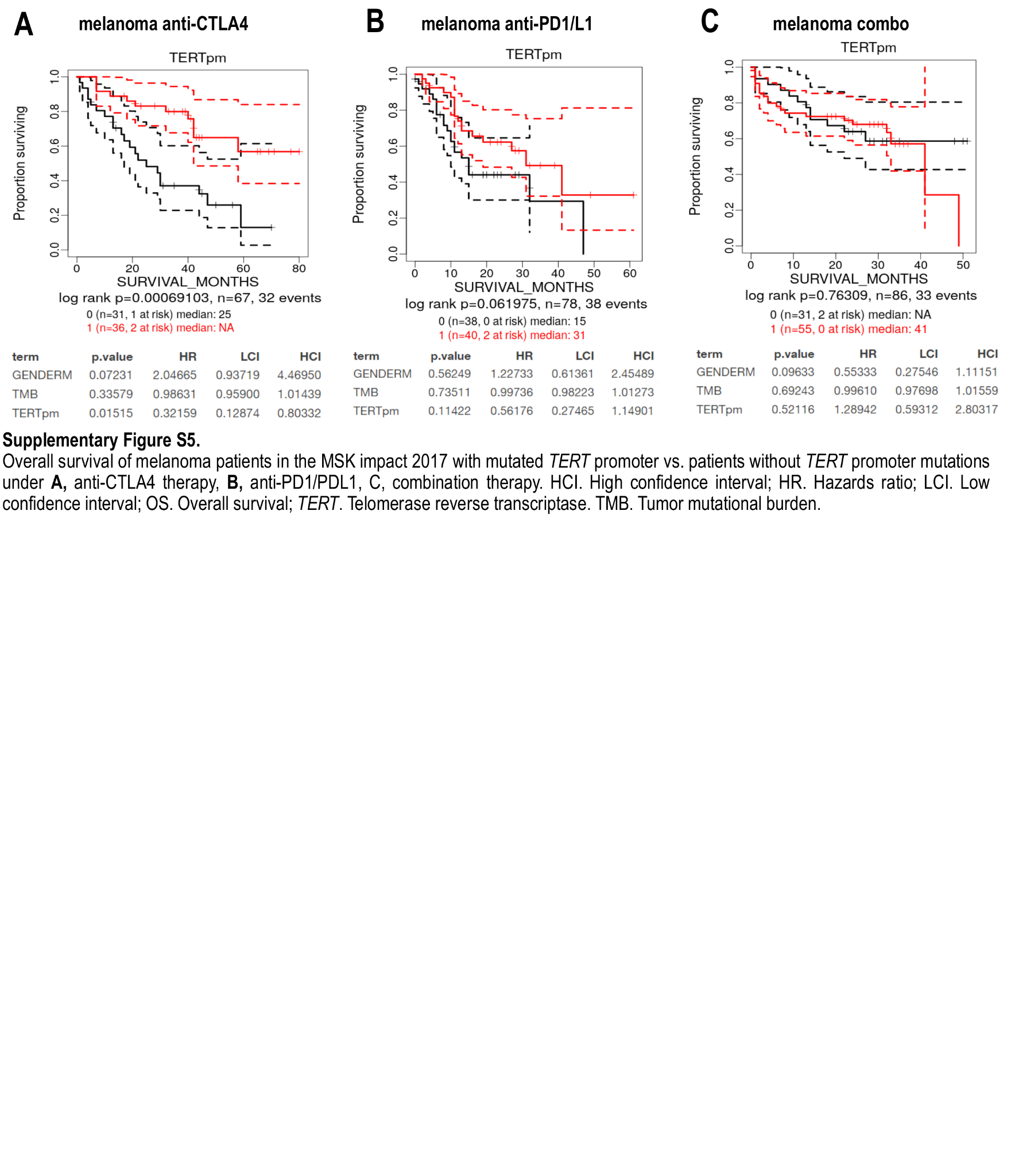


### S6 Fig. Univariate and multivariate survival analysis of reanalyzed melanoma MSK Impact 2017 subcohort. Overall survival of melanoma patients in the MSK impact 2017 with mutated *TERT* promoter^a^ vs. non mutated under (A) anti-CTLA4 therapy, (B) anti-PD1/PDL1, (C) combination therapy. HCI: high confidence interval; HR: Hazards ratio; LCI: low confidence interval; OS: overall survival; TERT: telomerase reverse transcriptase. TMB: tumor mutational burden.

^a^ Signature to get TERT promoter mutated patients: [,'TERT..MUT']=='Promoter'|[,'TERT..MUT']=='Promoter, D718N'|[,'TERT..MUT']=='E79K, Promoter'|[,'TERT..MUT']=='Promoter, E280K'|[,'TERT..MUT']=='Promoter, G463D'|[,'TERT..MUT']=='Promoter, H687Y'|[,'TERT..MUT']=='Promoter, K583N'|[,'TERT..MUT']=='Promoter, L477F'|[,'TERT..MUT']=='Promoter, P265Q'|[,'TERT..MUT']=='Promoter, Promoter'|[,'TERT..MUT']=='Promoter, Promoter, Promoter'|[,'TERT..MUT']=='Promoter, Q921*, Promoter'|[,'TERT..MUT']=='Promoter, Promoter, X885_splice'|[,'TERT..MUT']=='Promoter, R1097K, E440K'|[,'TERT..MUT']=='Promoter, R194*'|[,'TERT..MUT']=='Promoter, R315H'|[,'TERT..MUT']=='Promoter, S619F, Q569*'

#
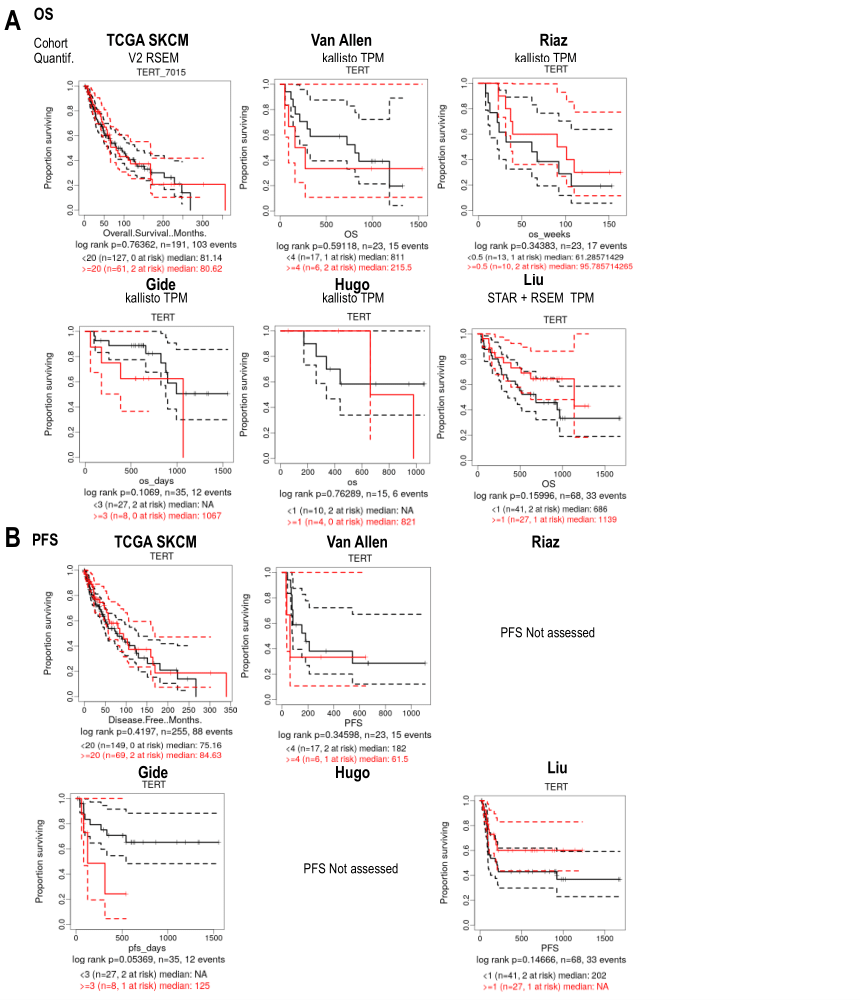


### S7 Fig. **Survival of *TERT* low vs. high patients with high CD4+ Th1 xCell signature.** (A) Patients with higher *TERT* expression with high CD4+ Th1 immune infiltration do not show altered OS and (B) PFS. Univariate p-values from logrank tests. Dashed: confidence intervals. The CD4+ T cell high subgroups were selected based on CD4+ Th1 signature over: SCKM: 0.04; Van Allen: 0.03; Riaz: 0.1, Gide: 0.02; Hugo: 0.01; Liu: 0.04. Note the varying thresholds for *TERT* expression in each cohort is due to the classification that the patient group is approximately one third patients with high *TERT* expression. Gide and Riaz cohorts subsetted for pre-therapy biopsies and SKCM for metastatic patients. OS: overall survival; PFS: progression-free survival; SKCM: skin cutaneous melanoma; TERT:telomerase reverse transcriptase; TPM: transcripts per million; Th1: T-helper type 1; xCell: Xcell cell types enrichment analysis.


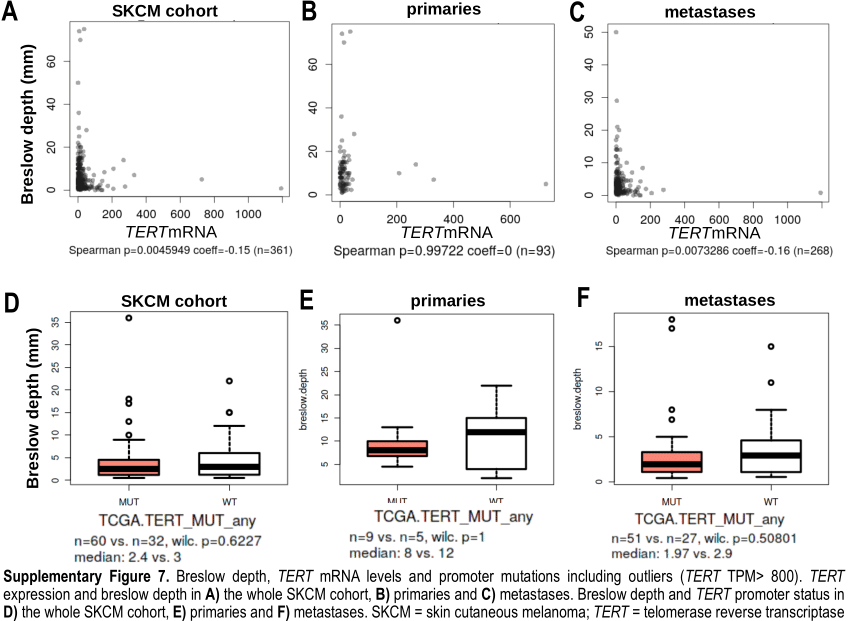


### S8 Fig. Breslow depth, *TERT* mRNA levels and promoter mutations including outliers (*TERT* TPM >800). Breslow depth of primaries and *TERT* expression measured in corresponding tumors of (A) the whole SKCM cohort, (B) in primary tumors and (C) in metastatic tumors. Breslow depth of primaries and *TERT* promoter mutations measured in corresponding tumors of (D) the whole SKCM cohort, E, in primary tumors and F, in metastatic tumors. SKCM: Skin cutaneous melanoma. *TERT*: Telomerase reverse transcriptase.


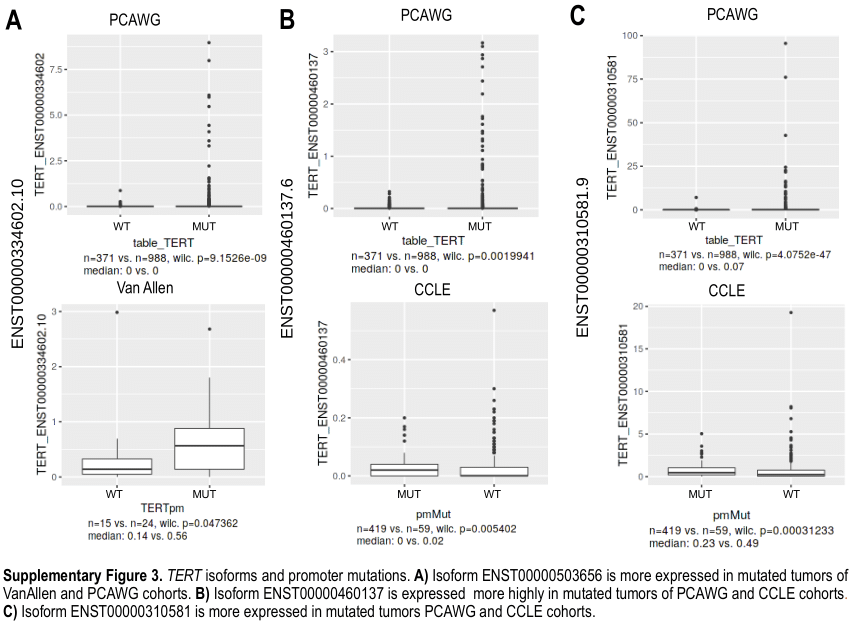


### S9 Fig. *TERT* isoforms with increased expression in the presence of promoter mutations. (A) Isoform ENST00000334602 (TERT-202) replicated in the Van Allen and PCAWG bulk tumor cohorts. (B) Isoforms ENST00000460137 (TERT-203) and (C) ENST00000310581 (TERT-201) replicated in PCAWG bulk tumors and CCLE cell lines. No isoform showed decreased expression with promoter mutations. CCLE: cancer cell line encyclopedia; Mito data: mitochondrial data available; PCAWG: pan-cancer analysis of whole genomes.


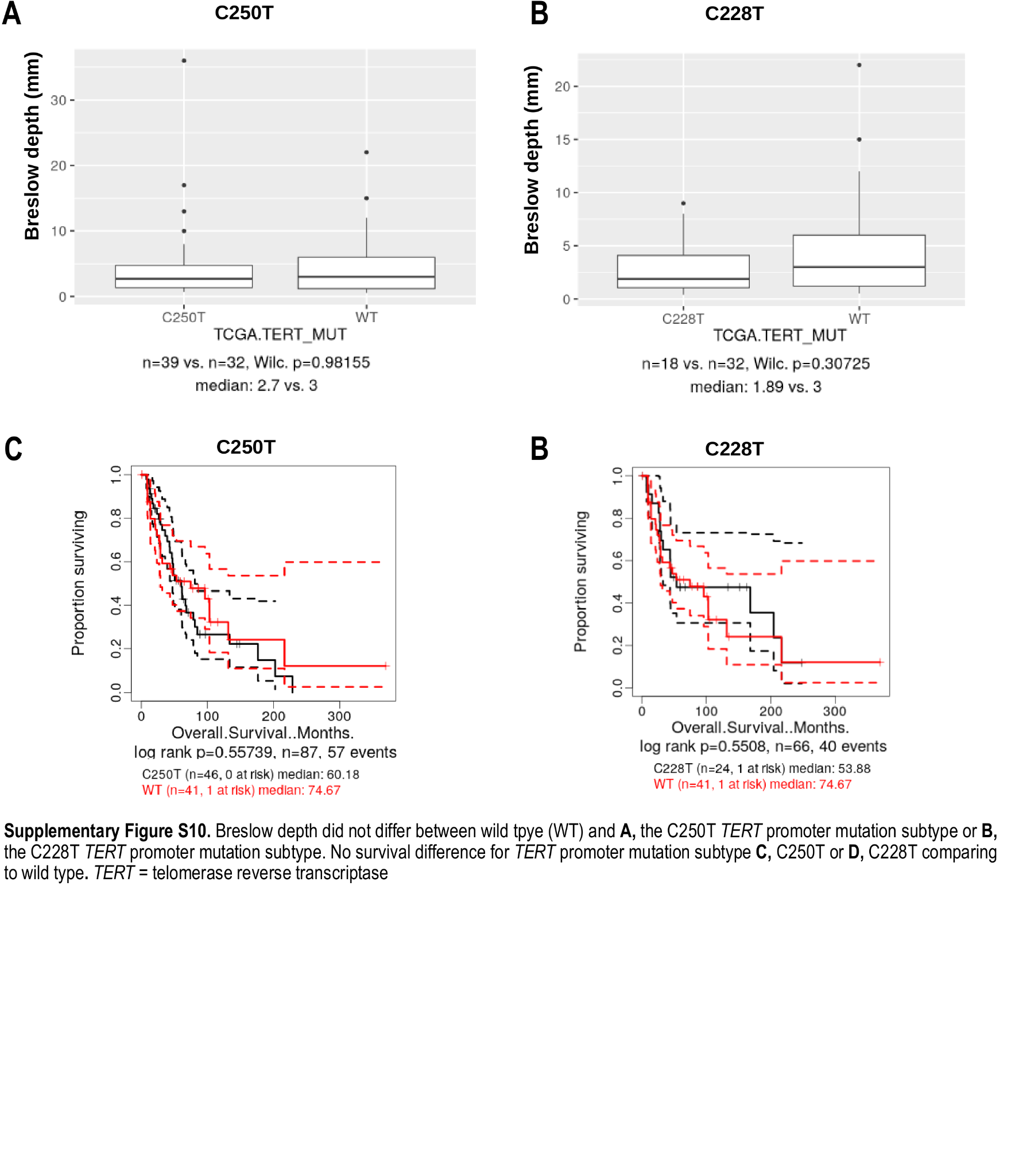


### S**10 Fig**. **Breslow depth and survival analysis for different *TERT* promoter mutation subtypes C250T and C228T.** Breslow depth did not differ between wild-type (WT) and (A) the C250T or (B) the C228T *TERT* promoter mutation subtype. No survival difference for *TERT* promoter mutation subtype (C) C250T or (D) C228T mutated or non-mutated. *TERT* = telomerase reverse transcriptase.

##

## Supplementary References

1. [Cancer Genome Atlas Network. Genomic Classification of Cutaneous Melanoma. Cell. 2015;161: 1681–1696.](http://paperpile.com/b/vLfZxA/0xxOg)

2. [Gide TN, Quek C, Menzies AM, Tasker AT, Shang P, Holst J, et al. Distinct Immune Cell Populations Define Response to Anti-PD-1 Monotherapy and Anti-PD-1/Anti-CTLA-4 Combined Therapy. Cancer Cell. 2019;35: 238–255.e6.](http://paperpile.com/b/vLfZxA/k99oX)

3. [Hugo W, Zaretsky JM, Sun L, Song C, Moreno BH, Hu-Lieskovan S, et al. Genomic and Transcriptomic Features of Response to Anti-PD-1 Therapy in Metastatic Melanoma. Cell. 2016. pp. 35–44.](http://paperpile.com/b/vLfZxA/l3HOs)

4. [Liu D, Schilling B, Liu D, Sucker A, Livingstone E, Jerby-Arnon L, et al. Integrative molecular and clinical modeling of clinical outcomes to PD1 blockade in patients with metastatic melanoma. Nat Med. 2019;25: 1916–1927.](http://paperpile.com/b/vLfZxA/xxLJj)

5. [Riaz N, Havel JJ, Makarov V, Desrichard A, Urba WJ, Sims JS, et al. Tumor and Microenvironment Evolution during Immunotherapy with Nivolumab. Cell. 2017;171: 934–949.e16.](http://paperpile.com/b/vLfZxA/YRihg)

6. [Van Allen EM, Miao D, Schilling B, Shukla SA, Blank C, Zimmer L, et al. Genomic correlates of response to CTLA-4 blockade in metastatic melanoma. Science. 2015;350: 207–211.](http://paperpile.com/b/vLfZxA/7VQA7)

7. [ICGC/TCGA Pan-Cancer Analysis of Whole Genomes Consortium (2020). Pan-cancer analysis of whole genomes. Nature. 2020;578: 82–93.](http://paperpile.com/b/vLfZxA/ut2HZ)

8. [Ghandi M, Huang FW, Jané-Valbuena J, Kryukov GV, Lo CC, Robert McDonald E, et al. Next-generation characterization of the Cancer Cell Line Encyclopedia. Nature. 2019;569: 503–508.](http://paperpile.com/b/vLfZxA/qtLUQ)

9. [Jerby-Arnon L, Shah P, Cuoco MS, Rodman C, Su MJ, Melms JC, et al. A Cancer Cell Program Promotes T Cell Exclusion and Resistance to Checkpoint Blockade. Cell. 2018;175: 984–997.e24.](http://paperpile.com/b/vLfZxA/nRyJM)

10. [Li H, Li J, Zhang C, Zhang C, Wang H. TERT mutations correlate with higher TMB value and unique tumor microenvironment and may be a potential biomarker for anti-CTLA4 treatment. Cancer Med. 2020;9: 7151–7160.](http://paperpile.com/b/vLfZxA/Fu2jB)

11. [Samstein RM, Lee C-H, Shoushtari AN, Hellmann MD, Shen R, Janjigian YY, et al. Tumor mutational load predicts survival after immunotherapy across multiple cancer types. Nat Genet. 2019;51: 202–206.](http://paperpile.com/b/vLfZxA/snUWd)

12. [Klughammer J, Kiesel B, Roetzer T, Fortelny N, Nemc A, Nenning K-H, et al. The DNA methylation landscape of glioblastoma disease progression shows extensive heterogeneity in time and space. Nat Med. 2018;24: 1611–1624.](http://paperpile.com/b/vLfZxA/1mZWR)

13. [Bray NL, Pimentel H, Melsted P, Pachter L. Near-optimal probabilistic RNA-seq quantification. Nat Biotechnol. 2016;34: 525–527.](http://paperpile.com/b/vLfZxA/FF4Es)

14. [Sieverling L, Hong C, Koser SD, Ginsbach P, Kleinheinz K, Hutter B, et al. Genomic footprints of activated telomere maintenance mechanisms in cancer. Nat Commun. 2020;11: 733.](http://paperpile.com/b/vLfZxA/9Gx75)

15. [Li T, Fu J, Zeng Z, Cohen D, Li J, Chen Q, et al. TIMER2.0 for analysis of tumor-infiltrating immune cells. Nucleic Acids Res. 2020;48: W509–W514.](http://paperpile.com/b/vLfZxA/WwGxp)

16. [Sturm G, Finotello F, Petitprez F, Zhang JD, Baumbach J, Fridman WH, et al. Comprehensive evaluation of transcriptome-based cell-type quantification methods for immuno-oncology. Bioinformatics. 2019;35: i436–i445.](http://paperpile.com/b/vLfZxA/4aVjj)
