# Supplementary material for: TERT expression is associated with metastasis from thin primaries, exhausted CD4+ T cells in melanoma and with DNA repair across cancer entities
Source: PLoS One. 2023 Jul 7;18(7):e0281487. doi: 10.1371/journal.pone.0281487 (PMC10328343; doi:10.1371/journal.pone.0281487)
Supplement: Attachment — Submitted filename: Response to Reviewers.docx [file pone.0281487.s010.docx]

## Rebuttal letter

### **Replies to comments of editor**

**PLOS ONE's style requirements**. Changes according to style requirements have been made.

**BioRender Licence.** We want to clarify that we hold an academic license for BioRender and have therefore added "This figure was created with BioRender.com" to Figures 1,2,3 and S1. We uploaded the corresponding permissions for each figure see “Permissions_Figures.zip”. We hope this will clear up any confusion about the source of our figures.

**Concern**. Consider discussing if mutation in TERT could have any similar impact as expression and discuss the same with melanoma and others including thyroid by citing these articles PMID: 23766237; PMID: 24617711; PMID: 25024077.

**Author response:**

In general, *TERT* promoter mutations, along with methylation and copy number gain, provide one explanation for increased *TERT* expression in cancer. Therefore, *TERT* promoter mutations could have the same effect in this setting. However, we also investigated which pathways are associated with *TERT* promoter mutations or *TERT* expression by analyzing associated gene expression. Here, *TERT* expression revealed a pattern of DNA repair-related genes, but no such clear pattern for *TERT* promoter mutations. We addressed this in our discussion starting with "Also beyond melanoma, co-expression of *TERT* and DNA repair genes was shared in various cancer entities including GBM, however, no common gene expression patterns were observed for *TERT* promoter mutations...". We believe that the underlying causes of these differences could be an interesting topic for the future.

We agree with the editor that the analysis of *TERT* promoter mutations and *TERT* expression may be especially relevant in thyroid tumors and added a discussion about thyroid tumors together with the suggested references. We thank the editor for raising up this interesting connection.

We would like to thank the editorial team and the reviewers for their valuable comments and in-depth review of our manuscript. We are convinced that the critiques have had a great impact on the strengthening of the quality of our work. We have carefully addressed all the criticisms, especially those of reviewer #2, in the following sections.

### **Replies to comments of reviewer #1**

**General comment:** It would help to appreciate the importance of the identified TERT mRNA gene associations and the directions (up/down regulated) better if the authors could add the corresponding gene expression levels (raw expression counts normalized to logCPM (counts per million)) and the logFC (fold changes).

**Author response:**

As Table 3 was based on the integration of up to nine different cohorts, we used expression measures in TPM as most of the studies did. We did not see a need to transform the data to logCPM and left them in TPM to have a straightforward integration with e.g., the Liu cohort, which we could only access in TPM.

For the reporting of the directions (up/down regulated) there is a column in Table 3 labeled "Consistent up or downregulated". This column highlights whether a gene is positively correlated with *TERT* mRNA expression (up) or negatively correlated with *TERT* mRNA expression (down) in all of the cohorts. To make this clearer, we have extended the footnote in Table 3. As we used correlation analysis instead of comparing two groups (low/high *TERT*) we do not provide fold changes, however, have included S7 Table, which contains the original results for each cohort used in the analysis. In S7 Table, the strength of the correlation can be assessed by looking at the correlation coefficient for each gene.

### **Replies to comments of reviewer #2**

**General comment:** In this manuscript, Kuhn et al. claimed that TERT alterations were not consistently associated with survival in melanoma cohorts under immune checkpoint inhibition. The presence of CD4+ T cells increased with TERT expression and correlated with the expression of exhaustion markers. While the frequency of promoter mutations did not change with Breslow thickness, TERT expression was increased in metastases arising from thinner primaries. Increased TERT expression was related to mitochondrial DNA stability and nuclear DNA repair during chromothripsis (PCAWG cohort) and under hypoxic conditions (PCAWG and SKCM cohorts). This should be a clear story, but authors tried to fit all the data into one story and made it so hard to understand and audiences were keeping lost in the irrelevant description.

**Author response:**

We thank the reviewer for the critical discussion of our manuscript.To make the manuscript and the comprehensive analysis of *TERT* changes clearer to the readers we restructured several parts of the manuscript accordingly.

In particular, we tried to make our story more transparent to the audience by rephrasing the abstract. We emphasize that our findings, particularly on *TERT* expression, add to the knowledge of non-canonical functions in melanoma metastasis and DNA repair. Finally, we have linked *TERT* expression to clinical relevance for immune checkpoint inhibition by discussing its potential influence on survival (which we ruled out) and potentially dysfunctional CD4+ T cells (which are still in the game). We have also removed some additional cohort information to make the abstract more readable. Throughout the manuscript we have reduced the text, e.g., removing the description of non-significant results in the section on intratumoral CD4+ T helper 1 cells (now just referring to the relevant Supplementary Table).

**Major point 1.** TERT promoter mutations were not always be associated with TERT expression in all cohorts. This made things much more complicated and confusing. If some mutations led to high TERT expression, while other mutations led to low TERT expression or irrelevant, authors should take this into account and some non-significant differences may actually significant.

**Author response:**

In three of the four cohorts studied (where *TERT* promoter mutation status was available), *TERT* promoter mutations were associated with increased *TERT* expression. We agree with the reviewer that the types of promoter mutation could have a different effect on *TERT* expression and now additionally tested these. However, individual mutations were only available for the SKCM and CCLE cohorts. Indeed, in both cohorts, C228T is the only mutation with significantly increased *TERT* expression (see S5D Table). We added these information in the discussion section together with S5D Table, S10 Fig shown here again:

**Additions to Table S5D: Difference between the different subtypes of *TERT* promoter mutations (in SKCM and CCLE) on *TERT* expression compared with wild type (WT).**

| **CCLE (across entities)** | **SKCM** |
| --- | --- |
| **C228T** (n=59) vs. WT (n=419) (**p=2.76e-5**, 2.28 vs. 1.04 TPM *TERT* expression) | **C228T** (n=25) vs. WT (n=46) (**p=0.0008**, 14.47 vs. 6.84 TPM *TERT* expression) |
| **C250T** (n=13) vs. WT (n=419) (n.s.) | **C250T** (n=41) vs. WT (n=46) (n.s.) |
| Too few tumors for: CC228TT (n=1), CC242TT (n=5), C228A (n=2), A161C (n=2) | Too few tumors for: C228T/C229T (n=1), C250T/C229T (n=2) |

Despite the fact that C228T is the second most common promoter mutation in melanoma (SKCM), it seems to have a stronger effect on *TERT* expression. We also tested whether the analyses of *TERT* promoter mutation with respect to Breslow depth changed when looking at the subtypes individually. None of the most common *TERT* promoter mutations subtypes showed a significant difference (S10A/B Fig, insufficient data to test mitochondrial mutations per individual mutation).

There were also no significant differences for survival (S10C and S10D Fig). Therefore, the results presented in our manuscript have not changed. However, we strongly agree with the reviewer that the effect subtypes of *TERT* promoter mutations should be taken into account in future research. This should be possible as the assessment of mutation types is currently part of routine testing.

**S10 Fig. Breslow depth and survival analysis for different TERT promoter mutation subtypes C250T and C228T.**

Breslow depth did not differ between wild type (WT) and **A,** the C250T *TERT* promoter mutation subtype or **B,** the C228T *TERT* promoter mutation subtype. No survival difference for *TERT* promoter mutation subtype **C,** C250T or **D,** C228T compared to wild type. *TERT*= telomerase reverse transcriptase.

**Major point 2.** It is so hard to understand the results parts and figures. They seem completely separated. I can’t even find where the figures were mentioned in the result parts (fig. 1B was even only in the discussion part). My suggestion is following the figures, which are much clearer than the results description.

**Author response:**

We appreciate the reviewer's suggestions regarding the structure of the results section. We have therefore made some contextual and structural improvements to make it clearer and more coherent:

An explanation at the beginning of the Results section outlines the order of each section, i.e. first *TERT* promoter mutations then *TERT* expression. In addition, we have provided a short introduction for each results section to provide a better understanding of the investigations.

Furthermore, we have made some structural changes to improve the presentation of the findings: Firstly, we have added a clear explanation of the findings presented in Figure 1B within the relevant section of the results. Secondly, we have restructured the results section to ensure that the order in which the findings are presented is consistent with that of their discussion. This includes *TERT* in melanoma subtypes first, followed by *TERT* with mutational load (3A), then DNA repair (3B), and finally *TERT* in mitochondria (3C). We believe that this reorganization will make it easier for readers to follow the logical flow of the findings. Lastly, we have rearranged the order of Figure 3 to correspond with the updated order of the results section. We hope these changes will help readers to process the findings presented in the figures more easily.

**Minor point 1.** There should be an abbreviation chart or explain every abbreviation when they appear first time.

**Author response:**

We agree with the reviewer and have therefore added an abbreviation table as suggested by the reviewer to make the abbreviations more accessible to any type of reader. We also added explanations for some immunotherapy-specific abbreviations, such as anti-CTLA4 and CD4+ T-cells.

**Minor point 2.** Page 6, single-cell RNA-seq data were mentioned. However, authors failed to describe the results.

**Author response:**

We apologize for any confusion caused by the results of our single-cell RNA-sequencing data. We would like to clarify what we did and where we addressed the results in our manuscript to address this issue:

First, we performed an overlap of the resulting genes associated with *TERT* expression from the bulk RNA-seq data with the tumor cell-specific genes from the scRNA-seq data. We now added a more specific description and the origin of the results in the manuscript in the results section (“S7A and S7H Table”). However, we want to point out that there is an additional column in Table 2 for scRNA data, which we also described. This shows that we have confirmed almost all of the genes found in the bulk RNA-seq data are predominantly expressed in malignant tumor cells.

Secondly, we conducted an enrichment analysis (last paragraph) with the top 100 genes co-expressed with *TERT* mRNA in the malignant tumor-cells from the scRNA data. We have now added more explicit information in the results section and information about the Supplementary Tables to further clarify our scRNA-seq results.

**Minor point 3.** Page 7, in first line, “was not associated with nonsynonymous”, is that a typo or the result in page 6 was wrong?

**Author response:**

We apologize for any confusion and would like to clarify that this whole paragraph is focusing on *mitochondrial* mutations, hence the mentioned nonsynonymous mutations are explicitly in mitochondria. As such, in the section titled "mutational load," *TERT* promoter mutations are found to be correlated with mutational load, but they are not correlated with "nonsynonymous *mitochondrial* mutations." To make this point more explicit, we have added the word "mitochondrial" a second time in the sentence.

Furthermore, in each paragraph of our manuscript, we address *TERT* promoter mutations (when available) and *TERT* expression. To make this clearer, we again want to emphasize the added paragraph in the beginning of the results section (also referred to in **major point 2**). In addition, we have restructured the content in a logical manner, starting with *TERT* promoter mutations and then discussing *TERT* expression.

We hope these clarifications provide a better understanding of our work.

**Minor point 4.** Discussion should mainly focus on authors’ data.

**Author response:**

We appreciate the reviewer's comment and value the importance of conciseness. However, we have tried to discuss all our findings in an appropriate way and since telomerase activation is a well-known phenomenon in cancer, there is plenty of existing literature. We tried to emphasize the relationship of what were new findings with respect to melanoma and non-canonical functions of TERT and hope the readability has improved.

### **Replies to comments of reviewer #3**

**General comment:** The manuscript-“TERT expression is associated with metastasis from thin primaries, exhausted CD4+ T cells in melanoma, and with DNA repair across cancer entities” by Kuhn et al, is an interesting study adding new insights into melanoma metastasis that is attributed to TERT expression. The manuscript is well-written, and the methodology is adequately described. The overall results presented in the manuscript are logically described. The choice of melanoma cohorts, cell lines as well as single-cell RNA seq data analysis look appropriate in context to TERT mutations. Overall, the results are clear and compelling with a well-structured possible role of TERT in mitochondrial and nuclear DNA stability.

I would recommend this manuscript for publication.

**Authors response:**

We thank the reviewers for their feedback on our manuscript and look forward to the opportunity to publish our results in PLOS ONE.
